# Supplementary material for: Expression of unfolded protein response genes in post-transplantation liver biopsies
Source: BMC Gastroenterol. 2022 Aug 10;22:380. doi: 10.1186/s12876-022-02459-8 (PMC9364610; doi:10.1186/s12876-022-02459-8)
Supplement: Supplementary file 1 — Additional file 1. Individual patient characteristics. [file 12876_2022_2459_MOESM1_ESM.docx]

**Additional file 1: Individual patient characteristics.**

| **Groups** | **Sex** | **Age (y)** | **Race** | **Indication for LT** | **Months from LT** | **ALT (U/L)** | **AST (U/L)** | **ALP (U/L)** | **Total Bilirubin**  **(mg/dL)** | **Steatosis** |
| --- | --- | --- | --- | --- | --- | --- | --- | --- | --- | --- |
| *AR* | M | 47 | White | Alcohol, A-1AD | 9.6 | 252 | 141 | 226 | 1.2 | None |
| *AR* | F | 20 | White | Wilson Disease | 4.6 | 68 | 27 | 54 | 0.5 | None |
| *AR* | F | 64 | White | NASH | 10.2 | 195 | 178 | 157 | 1 | None |
| *NR:HBR* | M | 27 | White | PSC/AIH | 0.8 | 302 | 127 | 366 | 12.2 | None |
| *NR:HBR* | F | 43 | White | PSC | 60.0 | 27 | 50 | 164 | 3.5 | None |
| *NR:HBR* | M | 65 | White | PBC | 71.5 | 165 | 134 | 261 | 7.4 | None |
| *NR:Mild* | M | 26 | Other | PSC | 105.1 | 31 | 35 | 130 | 0.5 | None |
| *NR:Mild* | M | 65 | White | Alcoholic | 119.1 | 20 | 18 | 87 | 1.5 | Mild |
| *NR:Mild* | M | 35 | White | Autoimmune | 6.5 | 84 | 48 | 68 | 0.7 | None |
| *NR:Mild* | F | 62 | White | Cryptogenic | 97.5 | 79 | 64 | 113 | 0.6 | None |
| *NR:Others* | F | 65 | Hispanic/Latino | Cryptogenic, HCC | 62.2 | 303 | 103 | 432 | 1.5 | Moderate |
| *NR:Others* | F | 58 | White | NASH | 57.5 | 22 | 24 | 303 | 0.6 | Mild |
| *NR:Others* | M | 25 | White | Biliary Atresia | 4.7 | 75 | 47 | 621 | 1.1 | None |
| *NR:Others* | F | 65 | White | PBC | 50.3 | 24 | 24 | 235 | 0.5 | None |
| *NR:Others* | F | 62 | White | PBC, HCC | 7.0 | 152 | 22 | 221 | 1.3 | None |
| *NR:Others* | M | 45 | White | PSC | 112.8 | 39 | 37 | 195 | 0.9 | None |
| *NR:Others* | F | 59 | White | PBC | 91.0 | 41 | 29 | 533 | 0.3 | None |
| *NR:Others* | M | 62 | Back | HCV | 35.3 | 105 | 40 | 191 | 1.2 | Mild |
| *NR:Others* | M | 43 | White | Cryptogenic | 142.5 | 124 | 74 | 532 | 1.5 | None |

***AR***: acute rejection

***NR:HBR***: non-rejection with hyperbilirubinemia (serum total bilirubin > 2.5 mg/dL)

***NR:Mild***: non-rejection; serum total bilirubin ≤ 2.5 mg/dL; ALT, AST and ALP ≤ 1.67x ULN

***NR:Others***: other non-rejection with serum total bilirubin ≤ 2.5 mg/dL; ALT, AST and ALP > 1.67x ULN
