# Supplementary material for: Expression of unfolded protein response genes in post-transplantation liver biopsies
Source: BMC Gastroenterol. 2022 Aug 10;22:380. doi: 10.1186/s12876-022-02459-8 (PMC9364610; doi:10.1186/s12876-022-02459-8)
Supplement: Supplementary file 3 — Additional file 3. Hepatic unfolded protein response gene expression correlated with serum AST. [file 12876_2022_2459_MOESM3_ESM.docx]

**Additional file 3: Hepatic unfolded protein response gene expression correlated with serum AST.** Graphs demonstrating the Pearson correlation between the serum AST levels and hepatic gene expression of the downstream targets of the XBP1, PERK and ATF6 pathways.
