# Supplementary material for: Expression of unfolded protein response genes in post-transplantation liver biopsies
Source: BMC Gastroenterol. 2022 Aug 10;22:380. doi: 10.1186/s12876-022-02459-8 (PMC9364610; doi:10.1186/s12876-022-02459-8)
Supplement: Supplementary file 4 — Additional file 4. Hepatic unfolded protein response gene expression correlated with serum total bilirubin. [file 12876_2022_2459_MOESM4_ESM.docx]

**Additional file 4: Hepatic unfolded protein response gene expression correlated with serum total bilirubin.** Graphs demonstrating the Pearson correlation between the serum total bilirubin levels and hepatic gene expression of the downstream targets of the PERK and ATF6 pathways.
