# Supplementary material for: Expression of unfolded protein response genes in post-transplantation liver biopsies
Source: BMC Gastroenterol. 2022 Aug 10;22:380. doi: 10.1186/s12876-022-02459-8 (PMC9364610; doi:10.1186/s12876-022-02459-8)
Supplement: Supplementary file 5 — Additional file 5. Hepatic bile acid metabolism gene expression correlated with serum liver chemistries. [file 12876_2022_2459_MOESM5_ESM.docx]

**Additional file 5: Hepatic bile acid metabolism gene expression correlated with serum liver chemistries.** Pearson correlation graphs demonstrated the hepatic expression of several bile acid metabolism genes correlated with serum ALT, AST, ALP and/or total bilirubin.
