# Supplementary material for: Expression of unfolded protein response genes in post-transplantation liver biopsies
Source: BMC Gastroenterol. 2022 Aug 10;22:380. doi: 10.1186/s12876-022-02459-8 (PMC9364610; doi:10.1186/s12876-022-02459-8)
Supplement: Supplementary file 6 — Additional file 6. Hepatic inflammation gene expression correlated with serum liver chemistries. [file 12876_2022_2459_MOESM6_ESM.docx]

**Additional file 6: Hepatic inflammation gene expression correlated with serum liver chemistries.** Pearson correlation graphs demonstrated the hepatic expression of several inflammatory genes correlated with serum ALT, AST and/or total bilirubin.
